# How Alfvén waves induce compressive flows in the neighborhood of a 2.5D magnetic null-point

**DOI:** 10.1038/s41598-020-70995-y

**Published:** 2020-09-24

**Authors:** S. Sabri, S. Vasheghani Farahani, H. Ebadi, S. Poedts

**Affiliations:** 1 Department of Theoretical Physics and Astrophysics, Faculty of Physics, University of Tabriz, P.O.Box 51666-16471, Tabriz, Iran; 2 Department of Mathematics, Center for mathematical Plasma Astrophysics, KU Leuven, Celestijnenlaan 200B, 3001 Leuven, Belgium; 3 Department of Physics, Tafresh University, Tafresh, 39518 79611 Iran; 4 Institute of Physics, University of Maria Curie-Skłodowska, ul. Radziszewskiego 10, 20-031 Lublin, Poland

**Keywords:** Space physics, Astronomy and planetary science, Mathematics and computing, Physics

## Abstract

The aim of the present study is to provide insight on the induced compressive perturbations together with the modifications of the environmental parameters in the course of Alfvén wave interaction with a solar magnetic null-point. The shock-capturing Godunov-type code PLUTO is used to solve the set of ideal magnetohydrodynamic equations. The nonlinear effects connected with an initial Alfvén pulse nearing a magnetic null point induces fast and slow magnetoacoustic waves with anti phase conduct. The induced current density and flows are independent of the local plasma-$\beta$ at the reconnection site. The induced inflows and outflows highly depend on the polarization. The inflows have a stronger effect compared to the outflows in both the *x* and *y* directions showing its peak in the *x*-direction. The dominant wave that couples to flows is the fast wave due to the in-phase harmony between perturbations of the compressive parameters and the fast wave. The induced current density possesses a steady orientation at the reconnection site which governs the diffusion or propagation of the waves. Induced perturbations by the nonlinear forces together with their back reaction on the Alfvén wave have a significant role in the current density excitation being responsible for the creation of inflows and outflows that are possible candidates for the creation of solar jets which has a significant contribution towards coronal seismology.

## Introduction

The Alfvén wave features itself in all aspects of plasma physics. The significance of the Alfvén wave roots in its nature where out of all the mgnatohydrodynamic (MHD) waves it is the only wave that in the linear regime does not perturb the plasma density and hence the neighboring sites. Nonetheless, in the nonlinear regime, the Alfvén wave indeed perturbs the plasma density where due to its wide range of direct and indirect effects plays influential on the concept of e.g., energy transfer^1–3^, turbulence^4^, nonlinear cascade^5,6^, wave conversion^7,8^, wave coupling^9,10^, and phase mixing^11,12^, etc.

In the context of the present study the Alfvén wave is studied due to its induction aspect. This is in a sense that the induced MHD waves together with the perturbations of the physical variables in the course of Alfvén wave interaction with a magnetic null-point^13^ is modeled. As told by its name, a magnetic null-point is the center of a magnetic site where the magnetic field is zero. Such a structure is famous since it is a magnetic reconnection site^14,15^, where even the reconnection may exhibit an oscillatory conduct^16,17^. It is worth stating that a vast number of magnetic null-points exist in the solar atmosphere which is due to the complexity of the magnetic flux distribution. Thus, tens and thousands of magnetic null-points may be present at an instance of time in the solar corona^18,19^, which spans from coronal rain observations in magnetic null-point topology^20^ to jet observations rooted in reconnection sites^21^. In a plasma medium, the magnetic field lines may break and rearrange building a concept named as magnetic reconnection. A reconnection site provides a foundation for studying various aspects of wave dynamics, especially the interaction of waves with the null-point itself. A reconnection site in the solar atmosphere hosts solar flares^22^ and coronal mass ejections^23^ which are strong enough to affect the Earth’s atmosphere.

The observation of reconnection jets^21,24^ provided motivation for carrying out the experimental setup of the present study to shed light on a possible mechanism for the creation of solar jets. The main actor in this regard is the Alfvén wave where the domain under consideration is a magnetic reconnection site^25^. As Alfvén waves are known to induce compressive perturbations in plasmas with transverse structuring^26,27^, it would be very instructive to work out its induced waves and perturbations in a homogeneous medium in the course of interaction with a null-point. Before proceeding, it is worth stating that the interaction of MHD waves with a magnetic-null point^28^ is accompanied by density accumulation, see e.g.^29,30,31,32^. The perturbations of the density spikes together with the back reaction of the induced perturbations on the initial nonlinear Alfvén wave would surly provide insight on the creation of perturbations and induced flows that may result as solar jets.

The primary aim of this work is to provide insight on the nonlinear dynamics of MHD waves nearing a 2.5D magnetic null point by carrying out numerical simulations based on the implementation of the MHD theory. An initial Alfvén wave is kicked towards the magnetic null point by taking in to account its potential forces connected with the nonlinear effects, which creates fast and slow magnetoacoustic waves, see also Refs.^32,33^. These induced waves propagate across the magnetic field lines and accumulate at the null point. The perturbations of the physical variables together with the modification of the environmental conditions due to the induced waves are highlighted together with their back reaction on the initial Alfvén pulse as they evolve is highlighted. In the proceeding section, the initial conditions of a reconnection site that would host wave interaction is presented before providing details of the experimental setup. The succeeding sections are to shed light on the induced fast and slow magnetoacoustic waves together with the induced flows and perturbations of the physical variables which results in modifying the plasma parameters of the reconnection site, where a brief discussion is presented regarding the evolution of the plasma characteristics which contribute towards coronal seismology.

## Configuration of a typical solar magnetic X-point encountering MHD waves

It is very probable for a magnetic null point to encounter MHD waves of all kinds. Thus, the MHD theory which is a macroscopic approach suits well for modeling such solar atmospheric events. Since in the solar corona the plasma density of the medium is around $10^8$ cm$^{-1}$ which is about six orders of magnitude lower than the solar photosphere^34^, it is not far from reality if the effects of solar gravity and plasma viscosity are neglected. This implies the ideal MHD equations

1 $$\begin{aligned} \rho \left[ \frac{\partial {\mathbf {V}}}{\partial t} + ({\mathbf {V}}\cdot \nabla ){\mathbf {V}}\right]= & {} \left( \frac{1}{\mu }\nabla \times {\mathbf {B}}\right) \times {\mathbf {B}}-\nabla P, \end{aligned}$$

2 $$\begin{aligned} \frac{\partial {\mathbf {B}}}{\partial t}= & {} \nabla \times ({\mathbf {V}}\times {\mathbf {B}}), \end{aligned}$$

3 $$\begin{aligned} \frac{\partial \rho }{\partial t}+\nabla \cdot (\rho {\mathbf {V}})= & {} 0, \end{aligned}$$

4 $$\begin{aligned} \frac{\partial P}{\partial t}+({\mathbf {V}}\cdot \nabla )P= & {} -\gamma P \nabla \cdot {\mathbf {V}}, \end{aligned}$$

where $\mathbf {\rho }$, ${\mathbf {V}}$, ${\mathbf {B}}$, and ${\mathbf {P}}$ respectively represent the mass density, plasma velocity, magnetic field, and plasma pressure. The constants $\mu =4\pi \times 10^{-7}\; {\hbox {Hm}}^{-1}$ and $\gamma = 5/3$ denote the magnetic permeability and the ratio of the specific heats. The sound speed is defined as

5 $$\begin{aligned} \mathbf {C_{{\mathrm {s}}}}= \sqrt{\frac{\gamma P}{\rho }}. \end{aligned}$$

The magnetic tension causes an elastic restoring force such that waves move along uniform magnetic field lines with the Alfvén velocity obtained by

6 $$\begin{aligned} \mathbf {V_{{\mathrm {A}}}}= \sqrt{\frac{B^2}{\mu \rho }}. \end{aligned}$$

Consider a magnetic null-point located at a static medium ($\mathbf {V_{0}} = 0$) with a magnetic field expressed as

7 $$\begin{aligned} {\mathbf {B}}= \frac{B_{0}}{L} (x,-y,0), \end{aligned}$$

where $B_{0}$ represents a characteristic magnetic field strength with *L* being the length scale for magnetic field variations. In the context of the present study the magnetic equilibrium structure is curl-free which includes a single null-point located at the origin. The magnetic field under consideration could be expressed in terms of a vector potential as

8 $$\begin{aligned} \mathbf {A_0} = \frac{B_{0}}{L} (0,0,xy). \end{aligned}$$

The specific quantitative values of the initial equilibrium density and sound speed are chosen consistent with the typical parameters of the solar coronal plasma respectively equal to $10^{-15}\; {\hbox {g/cm}}^3$ and $0.129\; \hbox {Mm/s}$, see Gruszecki et al.^41^.

## The experimental setup

The dynamics and energy transfer connected with Alfvén and magnetoacoustic pulses propagating towards a magnetic null-point are simulated. The various courses that these waves go through while encountering the null-point together with its outcome is observed in the context of MHD theory. MHD Eqs. (1)–(4) are solved numerically using the high-resolution shock-capturing code PLUTO, that is an appropriate and robust tool for studying nonlinear dynamics of magnetized fluids^35^. This code is appropriate for explicit time-dependent computations in three dimensions, where in the present study the Godunov method is implemented which allows taking into account the effects of entropy together with dissipation mechanisms. Note that three main parameters concerning the solar corona are taken as $\rho _{0} = 10^{-15} \frac{\text{gr}}{\text{cm}^{3}}$, $L_{0}= 10^{8}$ m, and $V_{0} = C_{A} = 10^{6} \frac{m}{s}$, which respectively represent the characteristic density, length scale, and velocity. Note that all other physical quantities of the system are made dimensionless. However, in the present study, ideal MHD equations are solved in 2.5D. The spatial coordinates in PLUTO code are generically labeled with $x_{1}$, $x_{2}$, and $x_{3}$ while their physical meaning depends on the value assigned to the geometry. Since Cartesian coordinates are implemented we have ${x_{1}, x_{2}, x_{3}} \equiv {x,y,z}$. It is worth noting that although the perturbations of three spatial components have been taken under consideration, but since the derivatives only with respect to the x and y components are considered, the approach is stated as 2.5 dimensions.

The magnetic field ${\mathbf {B}}$ is defined on the cell faces so how to maintain the restriction $\nabla \cdot {\mathbf {B}}=0$. The domain hosting the plasma medium possesses $(-10,10)\times (-10,10)\times (-10,10)\; \hbox {Mm}$ and $1400 \times 1400 \times 1400$ grid points. As the main focus is put on wave dynamics in the vicinity of a magnetic null-point, a stretched grid proves adequate to concentrate the majority of the grid points close to the null-point. Therefore, $1200 \times 1200 \times 1200$ grid points are set in the numerical domain $(-6,6)\times (-6,6)\times (-6,6)\; \hbox {Mm}$ with an effective resolution of $\delta x \approx \delta y \approx \delta z \approx 1/100\; \hbox {Mm}$. To state this clearer, the box size is set $100 \times 1200 \times 100$ with grid points through all three directions. There are 100 grid points in the physical volume $(-10,-6)$, 1200 grid points in the physical area $(-6,6)$, with 100 grid points in the physical volume (6, 10). The numerical grid size corresponds to 0.04 Mm in $(-10,-6)$ and (6, 10) and 0.01 in $(-6,6)$ that surrounds the magnetic null-point. The spatial resolution is much higher about the magnetic null point. The grid patch is constructed as $\delta x \approx \delta y \approx \delta z = \frac{(x_{R}-x_{L})}{N}\; \hbox {Mm}$ where $x_{R}$ and $x_{L}$ are the leftmost and rightmost points of the patch. The effect of the grid resolution could be examined by a test run with the lower spatial resolution. The result would show a similar action and evolution of waves for both cases.

**Figure 1:**
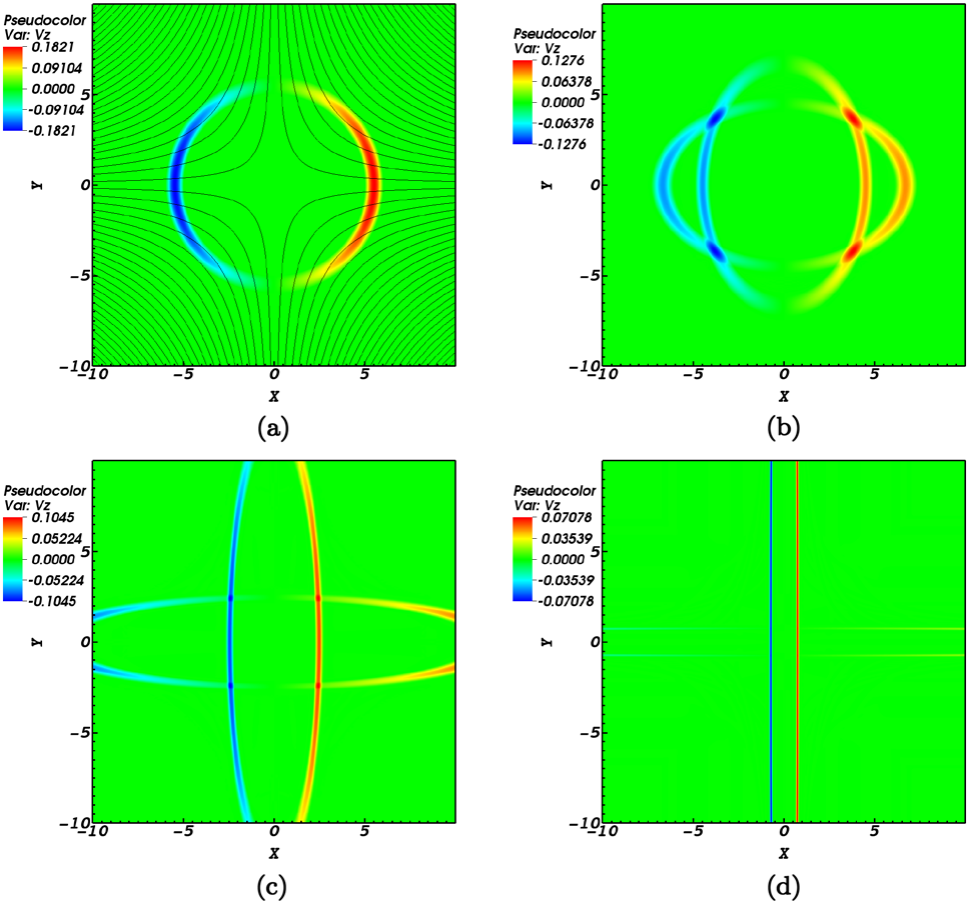
(**a**) shows the indicative lines of the equilibrium magnetic field structure and contours of the initial Alfvén pulse at $t=0$ normalized by $V_{0}= 10^{6}$ m/s. (**b**) represents the *z* component of the Alfvén pulse at $t=0.12$ s. (**c**,**d**) are snapshots of the pulse at $t=0.4$ s and $t=0.7$ s respectively. See also Ref.^1^.

To comply with the aims of the present study, consider a single circular wave pulse kicked in a static plasma medium at a finite distance from the magnetic null point where at time $t=0$ has a profile as of the form^1^

9 $$\begin{aligned} V_{{\mathrm {z}}}= A_0 \sin \left[ \pi \frac{\sqrt{x^2+y^2}-r_1}{r_0}\right] \frac{B_x}{\sqrt{(B_{{\mathrm {x}}})^2+(B_{{\mathrm {y}}})^2}}, \end{aligned}$$

with $V_{{\mathrm {x}}} = V_{{\mathrm {y}}} = 0$. The initial amplitude of the circular Alfvén pulse, $A_0$, is set equal to unity. It should be noted that spherically symmetric models are said to be the realistic models for astrophysical systems^36,37^. The typical values of the initial pulse are defined as $r_{0}=1 \, \hbox {Mm}$ and $r_{1}=5 \, \hbox {Mm}$. It is worth noting that the spatial selection for the circular Alfvén pulse (Fig. 1) resembles the initial state for the magnetoacoustic $(m=0)$ pulse of McLaughlin et al.^30^. This is due to the fact that the Alfvén pulse actually is independent of the azimuthal wave number^38,39^ making the azimuthal wave number equal to zero. Before proceeding it is worth providing a brief review of the experimental setups regarding the Alfvén wave interaction with magnetic null points in the solar atmosphere established prior to the present study; Galsgaard and co authors studied a helical Alfvén wave as it approached a 3D magnetic null-point, the system is governed by the Klein–Gordon equation. They concluded that as the Alfvén wave nears the magnetic null-point it spreads out. This is while the nonlinear induced magnetacoustic wave that they found, that its energy is much less than the initial Alfvén wave, wraps around the magnetic null-point^40^. By focusing on the propagation of magnetoacoustic waves McLaughlin and co-authors pictured how the wrapping process of magnetoacoustic waves is accompanied by the creation of a density spike at the null-point^29,30^. Thurgood and McLaughlin carried out a very interesting setup in 2.5D where they kicked an Alfvén pulse towards a magnetic null-point to found that Alfvén waves create transverse and longitudinal disturbances that transported little energy connected with the Alfvén wave towards the null-point due to the fact that weakly nonlinear effect where taken under consideration. Their emphasis was on the ponderomotive force in the presence of weakly nonlinear effects since their initial wave amplitude was considered relatively small. They reported an induced magnetoacoustic wave that carries part of the initial Alfvén wave towards the magnetic null-point where it experiences wrapping^12^. However what if a full description of nonlinear effects where taken under consideration. Therefore Sabri and co-authors carried out an experimental setup in order to study the induced fast and slow magnetoacoustic waves by the Alvén wave in the neighborhood of the null-point. Their aim was to measure the energy transfer rate due to induced fast and slow magnetoacoustic waves. They showed the shock formation regarding the initial Alfvén wave which induces large amplitude magnetoacoustic waves which is a signature of energy transfer from Alfvén to fast and slow magnetoacoustic that are to propagate towards the null-point. They reported that the fast wave experiences a sudden drop of energy transport which proves adequate for its energy release due to shock formation. This is while the slow wave experiences a gradual energy release, see Ref.^1^ for further details. In the present study the aim is to take a further step and highlight the back reaction of the induced fast and slow magnetoacoustic perturbations on the mother Alfvén wave. In this line we show how the nonlinear forces back react on the Alfvén wave and provide further insight on the sense in which the induced inflows and outflows contribute towards solar jet formation.

The analytic expression provided by Gruszecki et al.^41^ in the zero plasma-$\beta$ regime showed the dependence of the $m=0$ magnetoacoustic mode on the azimuthal wave number as it nears the null-point. This dependence proved adequate for the initial azimuthally symmetric $(m=0)$ mode to turn into an $m=2$ mode which is elliptic.

The Alfvén wave is a transverse wave that propagates along the direction of the magnetic field in a uniform medium. Fast magnetoacoustic waves propagate with an angle with respect to the magnetic field lines. However in the presence of transverse structuring magnetoacoustic waves experience refraction e.g.^42^ which guides them to propagate along the tube. This is in a sense that in the presence of magnetic field aligned density structuring in the medium, propagation of transverse waves along the magnetic field lines is expected^43^. In case of a magnetically twisted plasma medium with transverse structuring, the Alfvén wave is modified to the fast megnetoacoustic wave^38,39^ where its phase speed is determined by the Alfvén wave speed internal and external to the wave-guide^44^. The Alfvén speed is highly dependent on the plasma density; the nonlinear Alfvén wave propagation induces compressive perturbation due to the ponderomotive force^26,45,46^ which in turn perturbs the density. In this line induced variations of the magnetic field and plasma density would influence the local values of the the Alfvén speed. In fact the back reaction of the compressive perturbations would also affect the propagation of the Alfvén wave through the Cohen–Kulsrud equation^6^. This issue could also be considered as Alfvén wave self-interaction^47^. In this regard the nonlinear effects connected with the Alfvén wave propagation in the neighborhood of a magnetic null-point in the presence of transverse density structuring needs to be taken under consideration.

Figure 1 illustrates the velocity component, ${\hbox {V}}_{{\mathrm {z}}}$, of the Alfvén wave where the initial pulse has been intrinsically divided into two propagating waves. It is readily seen that due to the nature of Alfvén waves, the circular shape of the pulse deforms to follow the magnetic field lines. The Alfvén pulse propagates along the separatrices and accumulates along them. As the null-point is magnetic free, the Alfvén speed drops to zero at that very point, which disallows the presence of Alfvén waves at the null-point.

The propagation of nonlinear Alfvén waves induces compressive perturbations in transversely structured [e.g.^26^] and homogeneous [e.g.^1,12^] plasma medium. The outcome would be the creation of secondary waves. This is due to the nonlinear coupling of the transverse to longitudinal variables enabled by MHD equations. The total pressure perturbation generates slow and fast magnetosonic waves which are polarized in the direction perpendicular to the flux surfaces. The initial circular Alfvén pulse excites flows with two different velocity components, the component parallel to the magnetic field and the component perpendicular to the flux surface which indicates the presence of slow and fast magnetoacoustic waves^1,12^. The creation of the normal/perpendicular perturbations can be connected to the nonlinear ponderomotive force affiliated to the gradients of the perpendicular perturbed Alfvén speed^48^. On the other hand, the generation of the parallel flows may be associated to the nonlinear ponderomotive force that is induced by the gradients of parallel perturbed Alfvén speeds. The fact of the matter is that the nonlinear effects connected with the modification of the Alfvén wave^49–51^ which is subject to conditions in the domain of a magnetic null-point must be highlighted in order to contribute towards coronal seismology.

## Results and discussion

**Figure 2:**
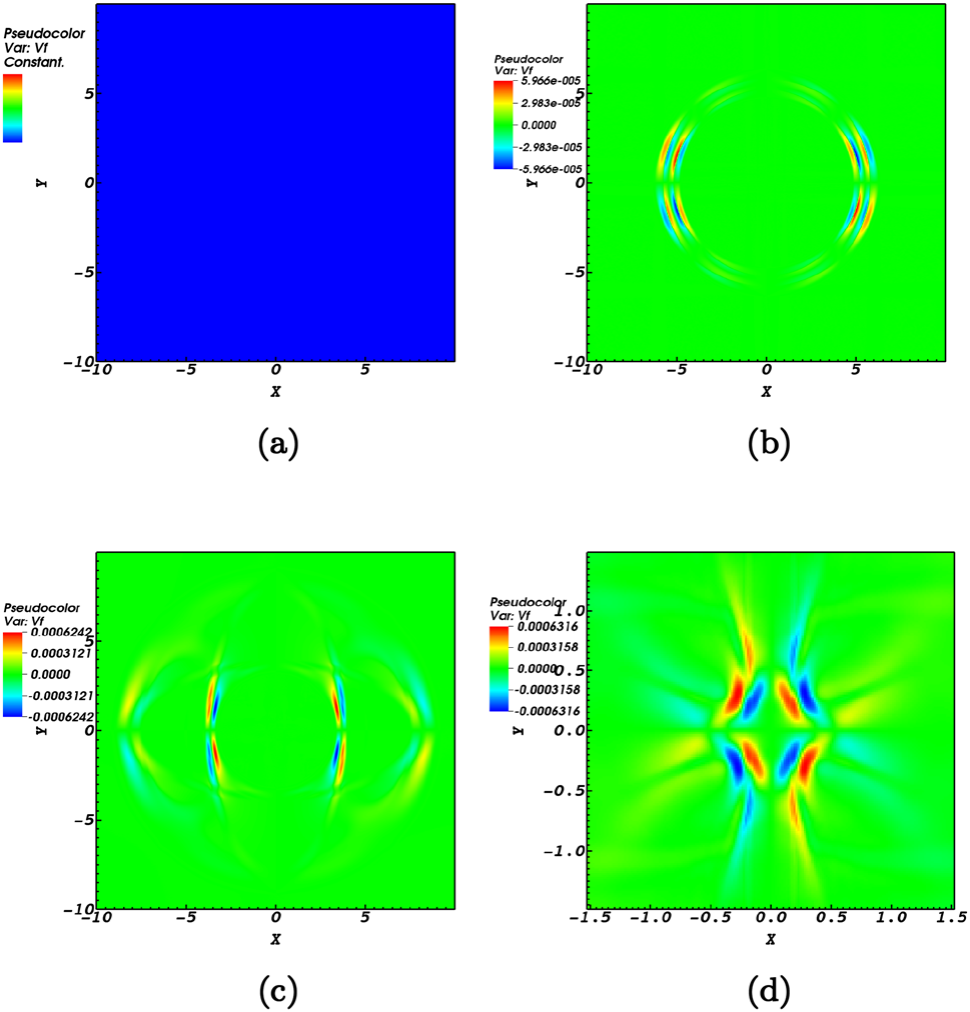
The numerical simulation of the induced fast wave represented by $v_\perp = v_{f}$ for snapshots at $t=0\;\hbox {s}$, $t=0.02\;\hbox {s}$, $t=0.2\;\hbox {s}$, and $t=1.72\;\hbox {s}$ normalized by $V_{0}= 10^{6}$ m/s. The propagating wave is observed to refract around the null-point where due to the finite plasma-$\beta$ effects passes through the null-point.

**Figure 3:**
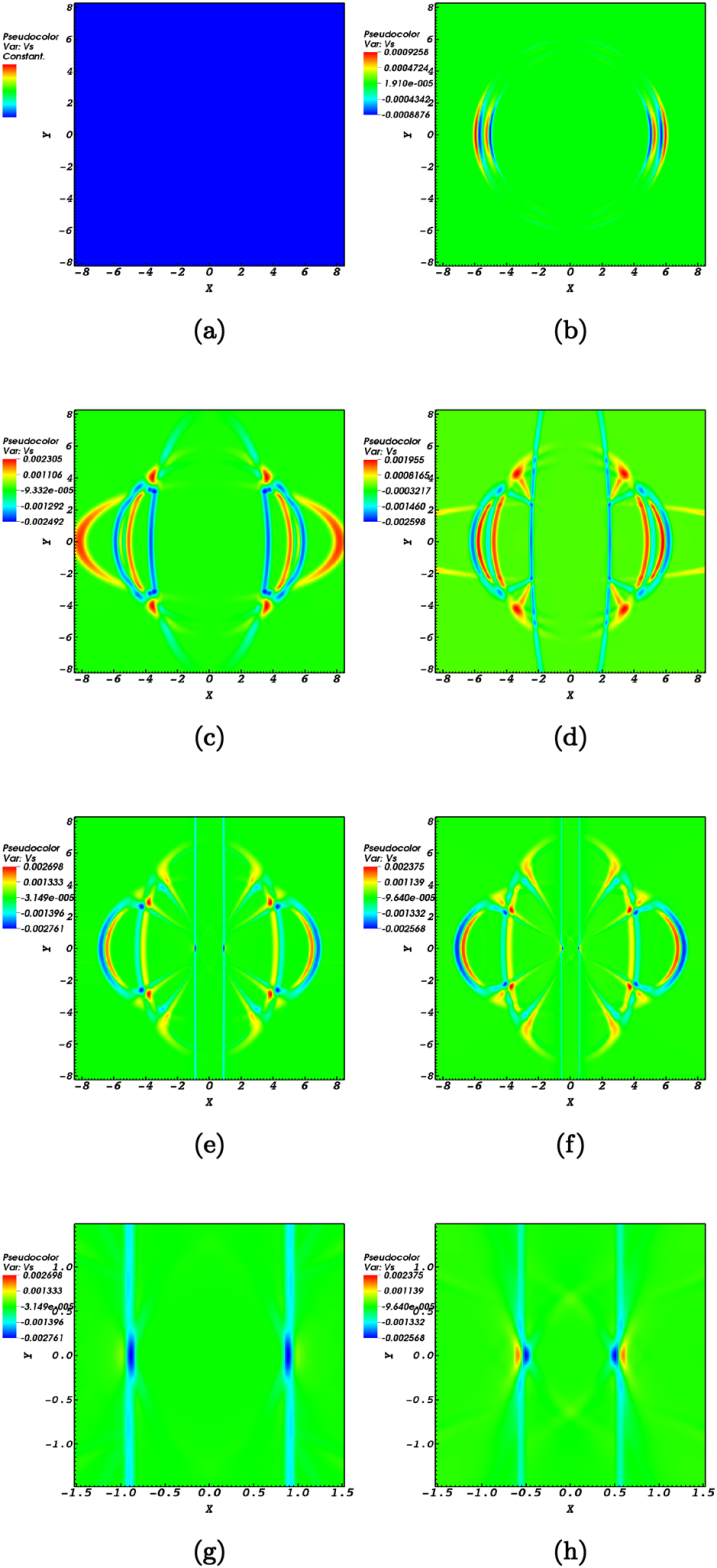
The numerical simulation of the induced slow wave represented by $v_\parallel = v_{s}$ for snapshots at $t=0\;\hbox {s}$, $t=0.02\;\hbox {s}$, $t=0.2\;\hbox {s}$, $t=0.4\;\hbox {s}$, $t=0.9\;\hbox {s}$, and $t=1.14\;\hbox {s}$ normalized by $V_{0}= 10^{6}$ m/s)shown in panels (**a**) till (**f**). The slow wave possesses a scaling behaviour where primarily focuses along the field line before wrapping around the null point. Panels **g**,**h** shed light on the slow wave refraction around the null point. In other hand, panels (**g**), (**h**) provide closer views of the slow wave at times $t=0.9\;\hbox {s}$, $t=1.14\;\hbox {s}$ to provide a close up view of how the slow wave spreads along the magnetic field lines before wrapping around the magnetic null point.

Magnetocoustic waves in homogeneous media could oscillate parallel or transverse with respect to the magnetic field lines. For coronal conditions, transverse oscillations are considered fast waves while longitudinal oscillations are considered slow waves. The propagation of the Alfvén wave nearing the null-point induces fast (Fig. 2) and slow (Fig. 3) magnetoacoustic waves.

### Induced fast waves

The perpendicular velocity profile that represents the induced fast wave propagating towards the magnetic null-point is presented in the four snap shots; $t=0\;\hbox {s}$, $t=0.02\;\hbox {s}$, $t=0.2\;\hbox {s}$, and $t=1.72\;\hbox {s}$ of Fig. 2. The velocity is defined as $v_\perp = ({\mathbf {V}}\times {\mathbf {B}}) \cdot z= v_{x}b_{y}-v_{y}b_{x}$. In Fig. 2a shows no magnetoacoustic profile due to the fact that at $t=0$ the only existing perturbation is the Alfvén pulse as shown in Fig. 1. Figure 2b which is a snapshot at $t=0.02$ shows that the fast magnetoacoustic wave has been created. For a medium where initially transverse structuring exists, the nonlinear effect due to transverse gradients in the Alfvén wave speed would create magnetoacoustic waves^48^. In the context of the present study, the equilibrium medium has no transverse structuring. But the nonlinearly induced perturbations due to the Alfvén pulse proves adequate for the induction of fast magnetoacoustic pulses. The initial form of the produced magnetoacoustic wave is observed to be circular just as of the primitive Alfvén pulse. Regarding a circular pulse it is instructive to read Ref.^12^.

A fast magnetoacoustic pulse experiences refraction around the magnetic null-point^29,30,41^ as pictured in the Fig. 2d. The blue and red colors of the pulse indicate anti-phase speeds in quarters 1 and 3 in comparison to quarters 2 and 4 which is a proof of the tendency of the initial $m=0$ mode to the $m=2$ mode, see also Ref.^41^. Due to the spatial non-uniformity created by the nonlinear Alfvén pulse, different parts of the wave move at different speeds which explains the refraction process as seen in Fig.  2. This wrapping process extracts a vast amount of the energy of the fast magnetoacoustic wave at the location of the null-point. A question that arises is whether this energy release is constant or possesses a scaling behaviour with respect to the altitude in the solar atmosphere in addition to local conditions and various circumstances. This requires the consideration of the finite $\beta$-plasma effects.

### Induced slow waves

The creation of slow magnetoacoustic waves which are longitudinal waves is also observed in the simulations conducted in the present study. Slow waves are highly subject to conditions in the solar atmosphere. The excitation of longitudinal waves in a zero plasma-$\beta$ medium is due to the longitudinal gradients in the Alfvén wave^48^. This is a pure nonlinear effect which needs to be further taken under consideration in finite plasma-$\beta$ conditions in the context of fast and slow magnetoacoustic wave interaction with magnetic null-points. Figure 3 shows the evolution of the parallel speed $v_\parallel$, i.e. a proxy of the induced slow magnetoacoustic wave traveling towards the null-point represented as $v_\parallel = {\mathbf {V}} \cdot {\mathbf {B}}=v_{x}b_{x}+v_{y}b_{y}$. It could be readily noticed from Figure 3a that at time $t=0\;$ there is no parallel velocity perturbation for the same reason as of the fast magentoacoustic pulse. The evolution of the parallel propagating wave is more complicated than that of the perpendicular wave. This is due to the fact that the plasma pressure gradient is the dominant driver for the slow wave which causes the slow wave speed to be dependent on the plasma-$\beta$.

Figure 3 proves the presence of the slow magnetoacoustic wave since parallel perturbations are non-zero towards the null-point. Nonetheless, the slow magnetoacoustic wave does not possess anti-phase propagation. The generated slow wave primarily spreads and accumulates along field lines before wrapping around the null-point according to the panel (d). Interestingly the slow wave experiences refraction around the magnetic-null point which could be readily noticed by Fig. 3 panels e,f. By taking a closer look at Fig. 3 panels g,h the slow wave wrapping could be better observed.

### Properties of the induced perturbations

**Figure 4:**
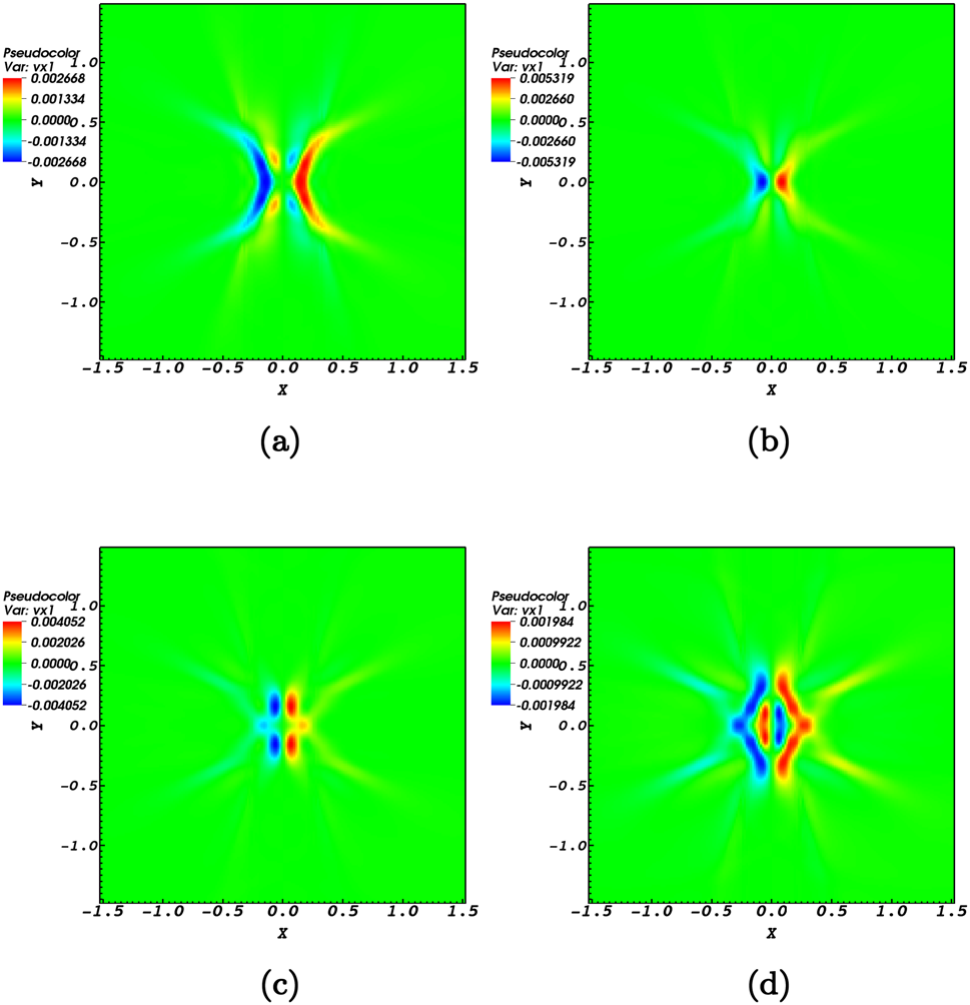
The propagation of $v_x$ towards the null-point normalized by $V_{0}= 10^{6}$ m/s for snapshots at times $t=1.46\;\hbox {s}$, $t=1.5\;\hbox {s}$, $t=1.56\;\hbox {s}$, $t=1.62\;\hbox {s}$. The blue colors indicate negative values of the velocity which proves the presence of inflows, while the red colors indicate positive values of the velocity which prove the presence of outflows.

**Figure 5:**
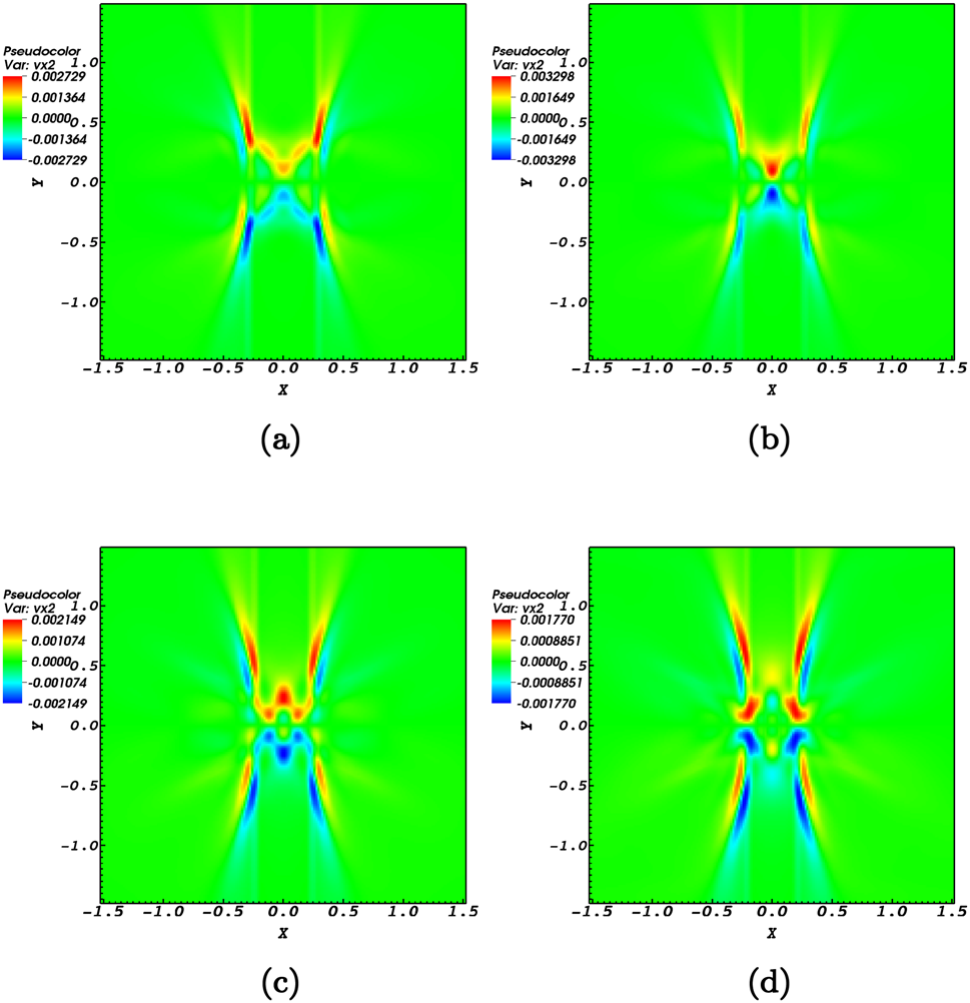
The propagation of $v_y$ towards the null-point normalized by $V_{0}= 10^{6}$ m/s for snapshots at times $t=1.46\;\hbox {s}$, $t=1.5\;\hbox {s}$, $t=1.56\;\hbox {s}$, $t=1.62\;\hbox {s}$. The blue and red colors respectively indicate the presence of inflows and outflows.

**Figure 6:**
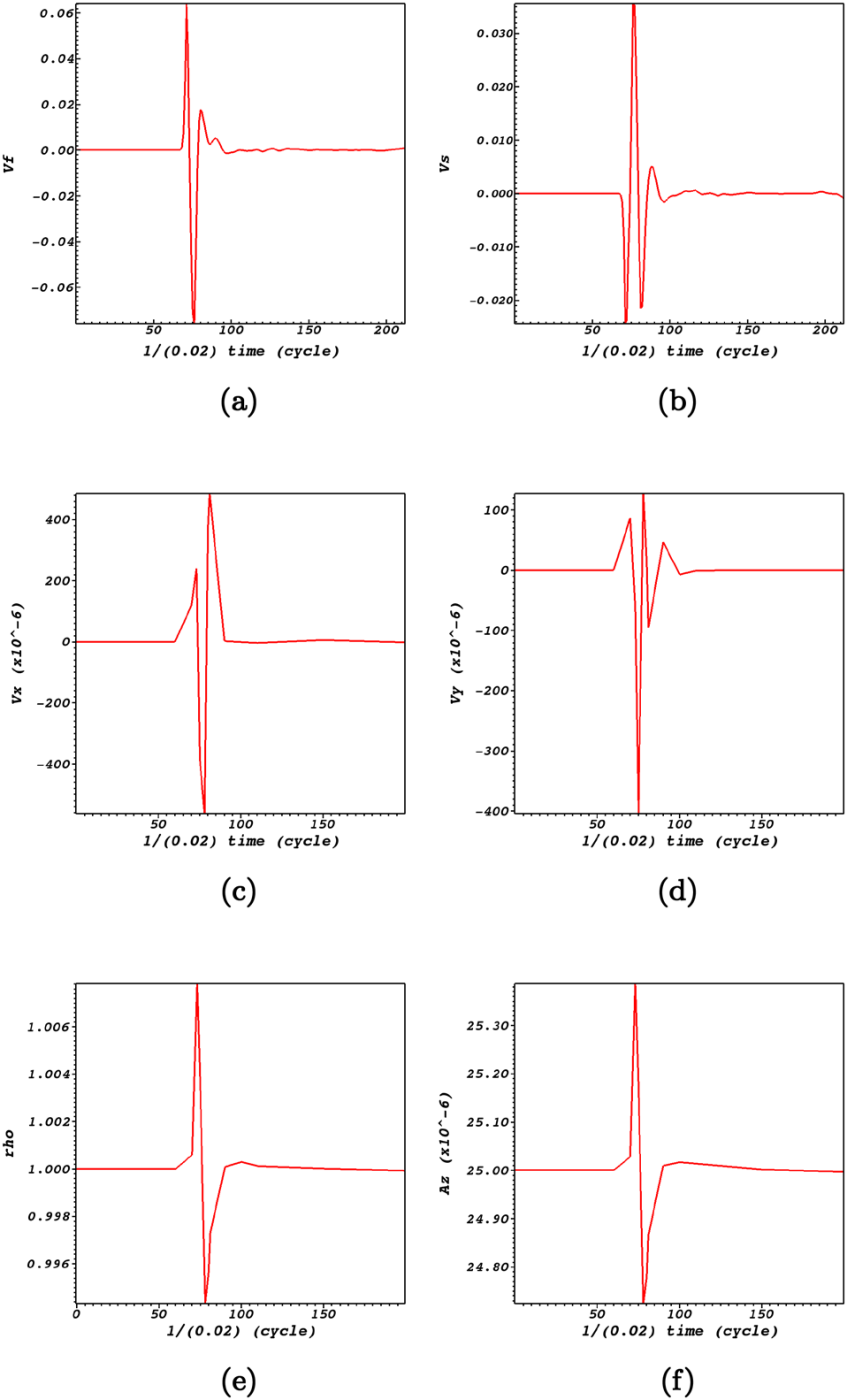
(**a**,**b**) respectively represent the time dependence of the induced fast and slow waves at the null-point. (**c**,**d**) show the time dependence of the *x* and *y* components of the induced flows normalized by $V_{0}= 10^{6}$ m/s at the null-point. (**e**,**f**) represent the time dependence of the mass density normalized by $\rho _{0}= 10^{-15}$ g/cm$^{3}$ and the vector potential at the null point (0,0,0).

The nonlinear Alfvén pulse is known to induce compressive perturbations in a medium with transverse structuring [e.g.,^26,27^]. In the context of the present study since the plasma medium is considered homogeneous the induced perturbations are checked by providing four snapshots associated with times $t=1.46\;\hbox {s}$, $t=1.5\;\hbox {s}$, $t=1.56\;\hbox {s}$, and $t=1.62\;\hbox {s}$ for the *x*-component of the induced velocity (Fig. 4) and the *y*-component of the induced velocity (Fig. 5). Perturbations of the compressive variables need also to be worked out. Perturbations of the the induced density perturbations $(\rho )$ together with perturbations of the velocity in the plane of the null-point, $v_{{\mathrm {x}}}$ and $v_{{\mathrm {y}}}$, are pictured with respect to time in Fig. 6. The fact of the matter is that the density is perturbed at the null-point which corresponds to the ponderomotive force that is connected to the variation of the absolute value of the magnetic field in the wave. This influences the gradient of the magnetic pressure that derives plasma oscillations and flows which are responsible for the density variations of the plasma as shown by Fig. 6. The outcome would present flows and compressive waves. Note that it has been suggested that Alfvén wave-driven outflows are responsible for solar winds^52,53^. As a matter of fact it is the induced perturbations that characterize the parameters and conditions of the magnetic null-point and higher its efficiency to contribute towards various physical events in the solar atmosphere. For instance, induced flows may be possible for jet formation in the neighborhood of a magnetic null-point^24^. The four snapshots in each of the Figs. 4 and 5 prove that both inflows and outflows are induced in the *x*-direction and *y*-direction at the neighborhood of the magnetic null-point. The blue colors indicate negative values of the velocity which proves the presence of inflows, while the red colors indicate positive values of the velocity which prove the presence of outflows. In Fig. 5 the snapshots at times $t=1.46\;\hbox {s}$, $t=1.5\;\hbox {s}$, $t=1.56\;\hbox {s}$, and $t=1.62\;\hbox {s}$ regarding the *y*-component of the induced flows, $v_y$, prove that both inflows and outflows are induced in the *y*-direction at the neighborhood of the magnetic null-point. The blue colors indicate negative values of the velocity which show the presence of inflows, while the red colors indicate positive values of the velocity which show the presence of outflows. By comparing the panels of Figs. 4 and 5 it could be noticed that the flows induced in the *y*-direction have a broader range compared with the inflows and outflows induced in the *x*-direction.

By comparing the time-signatures of the *x* and *y* components of the induced flows with the fast and slow magnetoacoustic waves at the null-point (Fig. 6), valuable information regarding their evolution could be extracted. It could be readily noticed from Fig. 6a,c that the induced flow velocity in the *x*-direction is in phase with the fast magnetoacoustic wave. This is while from Fig. 6b,d it could be noticed that the induced flow velocity in the *y*-direction is anti phase with the slow magnetoacoustic wave. However this anti phase behaviour turns to an in-phase behaviour as the slow wave wraps around the null-point, see the panels of Fig. 3. Figure 6e,f which indicate the time dependency of the density and the vector potential clearly satisfy the MHD force balance condition where the density presents its functionality on the vector potential. Figure 6e,f provide insight on the oscillatory behaviour of the null-point^30,54^ due to the oscillatory behaviour of the vector potential. It could be noticed that the oscillatory pattern is as of the density perturbations which itself is dictated by the induced fast magnetoacoustic wave, see Fig. 6a which proves adequate that the density is perturbed by the fast magnetoacoustic wave. The variation of plasma density leads to a variation of the sound and Alfvén speeds. This inhomogeneity of the characteristic speeds leads to an infinite set of eigenfrequencies or a continuum in the MHD spectrum. One of the consequences of the Alfvén continuum is the possibility of resonant absorption of the Alfvén wave energy at resonant layers where the local Alfvén frequency matches the frequency of the wave^55–58^.

**Figure 7:**
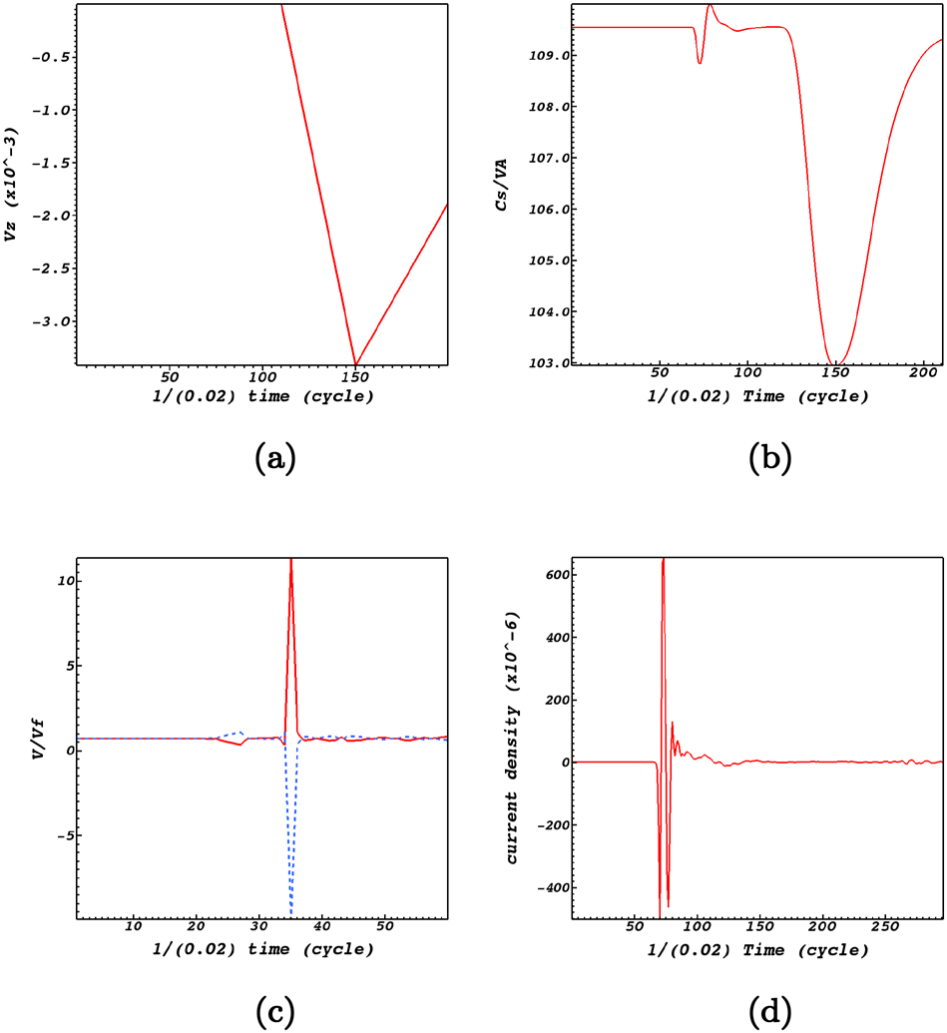
Evolution of the induced perturbations at the null-point. (**a**) regards the Alfvén wave. (**b**) regards the ratio of the acoustic and Alfvén speeds. (**c**) regards the ratio of the flows with respect to the fast wave, where the red curve is for $v_x$ and the blue curve is for $v_y$. (**d**) represents perturbations of the current density at the null point.

It must be stated that although induced perturbations are due to the nonlinear effects connected with the initial Alfvén wave, but these compressive perturbations back react on the Alfvén wave in addition to modifying the parameters and conditions of the magnetic null-point site. An important parameter in this regard is the plasma-$\beta$. Due to the efficiency of the sound speed at the null-point, fast and slow magnetoacoustic waves are able to pass through the null point. As there is no initial magnetic field at the null-point, and hence no local Alfvén speed, the null-point would host as an excellent domain to study the back reaction of the induced perturbations on the Alfvén wave itself. Figure 7a shows that at time-zero there is no Alfvén wave at any distance from the null-point. It could be readily noticed that the Alfvén perturbation which is connected with the nonlinear perturbations peaks at the null-point at times around 3 s. This is while the magnetoacoustic waves have already reached the null point, see Fig. 6. The back reaction is so how that enables the Alfvén pulse to modify the plasma-$\beta$ at the the null-point site.

It could be readily noticed by Fig. 7b that the ratio of the acoustic speed and the Alfvén speed which is a measure of the plasma-$\beta$ obtained by, $\beta \sim C_s^2/C_{\mathrm {A}}^2$, is non-zero which is proportional with $r^{-2}$ where *r* is the distance from the null-point. However, when the plasma-$\beta$ equals unity which is where we have $C_{{\mathrm {A}}}\approx C_s$ the mode conversion layer is achieved, this is where the low-$\beta$ fast wave can convert into both a high-$\beta$ fast and high-$\beta$ slow wave. It could also be readily noticed that variations of the plasma-$\beta$ follows an oscillatory pattern which causes variations of the plasma temperature. This induces outflows and inflows which are possible candidates for the creation of solar jets.

**Figure 8:**
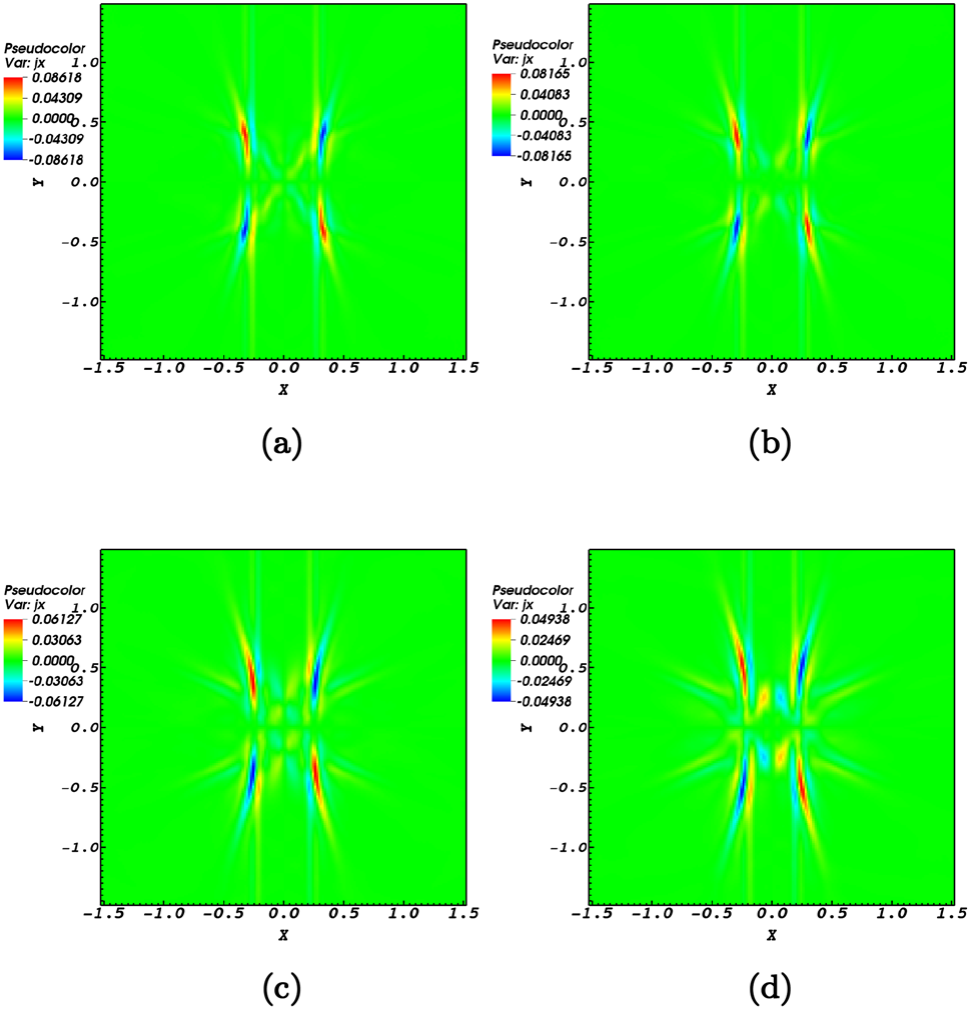
Contours of current density and magnetic structure at the null point (0,0,0) at times $t=1.46\;\hbox {s}$, $t=1.5\;\hbox {s}$, $t=1.56\;\hbox {s}$, $t=1.62\;\hbox {s}$.

Another aspect of picturing the evolution of the *x* and *y* components of the flow velocities is that they exactly act in a reverse manner with respect to each other (Fig. 7c). To state clearer, the induced flows connected with the fast magnetoacoustic waves would be outflows along the *x*-direction while at the same time experiencing inflows along the *y*-direction. Interestingly the peaks of the perturbations regarding fast and slow wave speeds are observed at the same instances at the null-point, see Fig. 7c. An aspect of the null-point is that fast magnetoacoustic waves convert to slow waves while passing the null-point. This is observable in Fig. 6 that minimum amount of fast wave is consistence with the maximum amount of slow wave that means fast wave converts to slow wave while crossing the null-point. Figure 7d represents the current density perturbations which is an aspect of the interaction of fast magnetoacosutic waves with the null-point, see also e.g., Refs.^30,41^ in this regard. It is instructive to figure out the behaviour of the current density with respect to the magnetic structure. Figure 8 shows snapshots of the electric current density at the magnetic null point. It illustrates that when the magnetoacoustic waves get to the magnetic null-point they excite a weak current density at the null point. Due to the destructive wave interference, the Alfvén wave excites small amplitude magnetoacoustic waves that lead to the creation of a weak current density. Therefore, it can not cause the X-point to collapse, but it is able to provide a steady configuration for the magnetic null-point.

Dynamics of the current density is pictured in Fig. 8. The fact of the matter is that in a magnetic null-point site it is the current density that dictates the diffusion or propagation of wave dynamics. Regarding a magnetic null-point accompanied by resistive dissipation, the current density couples with the magnetoacoustic wave and tends to regions where magnetic forces are present^59^. Since in the present study ideal conditions are taken under consideration it is instructive to provide snapshots of the current density perturbations at the null-point to see the dynamics of the current density. The four snapshots at time instance as for the other perturbations pictured in the present study are presented in Fig. 8. Although the current density possesses a steady orientation, but the forces connected to the nonlinear effects are responsible for the creation of inflows and outflows leading to the creation of solar jets. Another aspect of the nonlinearity connected to the Alfvén wave is the process of phase mixing which is present in an inhomogeneous medium due to the transverse gradients that the wave experiences across the background magnetic field which is a potential mechanism of coronal heating^60^. However the back reaction of the induced compressive perturbations on the Alfvén wave would act instead of the inhomogeneity in the background medium because of the presence of non-uniform background Alfvén speeds.

### Current density and plasma flows

Before proceeding to conclusions it is instructive to provide a parametric study consisting of macroscopic properties of plasmas in a one-fluid model. This model is a combination of hydrodynamic plus Lorentz effects for a one-fluid plasma together with the Maxwell equations for the electromagnetic fields. This provides insight on the current density generation regarding the flows. In this line the plasma momentum equation regarding the two important force factors are stated as

10 $$\begin{aligned} \rho \frac{\partial {\mathbf {V}}}{\partial t}= J\times B - \nabla P , \end{aligned}$$

where by operating the cross product ${\mathbf {B}}$ to the momentum equation, we obtain

11 $$\begin{aligned} {\left( B \times \rho \frac{\partial {\mathbf {V}}}{\partial t}\right) / (B^2)}= (B \times (J\times B))/ (B^2) - ( B \times \nabla P) / (B^2). \end{aligned}$$

Implement the $\mathbf {bac-cab}$ mathematic alliance and consider the perpendicular component of the current density with respect to the magnetic field, we have

12 $$\begin{aligned} J_{\perp } = {B \times \rho \frac{\partial {\mathbf {V}}}{\partial t}/ (B^2)} + (B \times \nabla P) / (B^2). \end{aligned}$$

**Figure 9:**
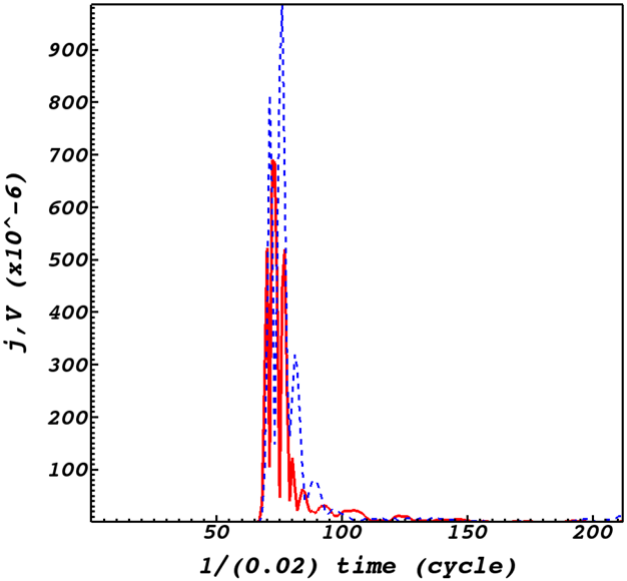
Time evolution of the current density (red curves) and plasma flow velocity (blue dashed curves) obtained by ${\mathbf {V}}= \sqrt{(V_{x})^{2} + (V_{y})^{2} }$, at the null point (0,0,0).

Equation (12) is an efficient equation that provides basis for analyzing MHD instabilities. The first term on the right hand side of Eq. (12) indicates the polarization current density while the second term defines the diamagnetic current density. The polarization current is the current excited by the sum of the currents due to the polarization flows. As for the diamagnetic flows, the current that is excited through them is called the diamagnetic current. Diamagnetic currents produce a magnetic field that reduces the magnetic field strength in proportion to the plasma pressure according to the Fig. 6. To understand the role of plasma flows on current densities, the magnitudes of the current density and plasma flow velocities (${\mathbf {V}}= \sqrt{(V_{x})^{2} + (V_{y})^{2} }$ ) is pictured in Fig. 9. It could be readily noticed that the shear flows have a significant role in the current density excitation. On the other hand, plasma exhibits the frozen-in behavior and therefore plasma flows lead to the accumulation of current density. In the end, it is worth noting that the current density is not only excited by nonlinear effects connected with circular polarized waves in fluids and plasmas, but also is excited by the vortex structures present in the context of Bose-Einstein condensation which may be considered as the fifth state of matter^62–64^ after the fourth state of matter which is plasmas. Spacecraft observations in the magnetotail connected to the Earth and laboratory experiments of magnetic reconnection have manifested the existence of electron current shear flows in current sheets^61^.

## Conclusions

In this study the interaction of induced perturbations together with their back reaction on the Alfvén wave in the course of interaction with a 2.5D magnetic null-point in the solar atmosphere is pictured. The numerical simulations are based on the nonlinear MHD theory carried out by the PLUTO code. The magnetic null-point structure, preconditions, equilibrium variables, and assumptions are studied under solar coronal conditions. Emphasis is on the induced compressive perturbations which constitute the fast and slow magnetoacoustic waves together with their back reaction on the initial Alfvén pulse in the course of interaction with a magnetic null-point. The scenario is as follows; as the Alfvén wave propagates along the magnetic field lines preserving its characteristics it accumulates along the separatrices. This is while the Alfvén wave produces nonlinear perturbations in the *x*–*y* plane directed towards the null-point in the perpendicular direction, normal to the flux surfaces. Although this wave is a transverse wave induced by the Alfvén wave, its propagation towards the null-point is independent of the Alfvén wave. This fast magnetoacoustic wave eventually accumulates at the null-point, see also Ref.^12^. Now what happens in the course of this interaction together with the succeeding effects and contributions towards coronal seismology is summerised as follows:

1. The induced fast and slow magnetoacoustic waves reach the null-point in an anti-phase pattern. Due to the initial non-zero plasma-$\beta$ conditions of the null-point, the slow wave speeds up as it approaches the null-point. The slow wave spreads along the field lines before accumulating on the null-point, while the fast wave refracts and crosses.
2. Although it might be thought that the local plasma-$\beta$ would affect the perturbations of the induced physical parameters. The induced perturbations of the compressive variables together with their back reaction on the initial Alfvén pulse are independent of the local plasma-$\beta$ at the reconnection site. The induced current density is also independent of the local plasma-$\beta$.
3. The strength of the induced inflows and outflows highly depends on the direction of the slow wave. The inflows are stronger than outflows in both the *x* and *y* directions. But, the inflows are almost double the strength of the outflows in the *x*-direction where the slow wave is propagating. This could be justified by the fact that the slow wave due to its longitudinal behaviour is acting only in the *x*-direction while the fast wave is dependent on the angle and acts on both directions. This causes a dominant effect in the direction of the slow wave (*x*-direction) .
4. The comparison of time variations of the induced fast and slow magnetoacoutic waves prove adequate for the domination of the induced fast magnetoacoustic waves on the oscillatory behavior of the compressive perturbations. Even the induced perturbations $V_x$ and $V_y$ follow the oscillatory behaviour of the fast magnetoacoustic wave. Hence the dominant wave that couples to flows is the fast wave.
5. The current density dictates the diffusion or propagation of wave dynamics at the reconnection site. For ideal conditions, the current density possesses a steady orientation. Nonetheless, the forces connected to the nonlinear effects are responsible for the creation of inflows and outflows leading to the creation of solar jets.

The effects connected with nonlinear forces regarding the propagation of Alfvén waves which induce compressive perturbations together with their back reaction on the mother Alfvén wave would construct a medium with nonuniform back ground Alfvén speeds. This phenomena would cause a homogeneous background medium to host features like phase mixing and other wave coupling effects that are signatures of homogeneous plasma medium.
